# Toward Experimental Evolution with Giant Vesicles

**DOI:** 10.3390/life8040053

**Published:** 2018-10-31

**Authors:** Hironori Sugiyama, Taro Toyota

**Affiliations:** 1 Department of Basic Science, Graduate School of Arts and Sciences, The University of Tokyo, 3-8-1 Komaba, Meguro-ku, Tokyo 153-8902, Japan; h-sugiyama@g.ecc.u-tokyo.ac.jp; 2 Universal Biology Institute, The University of Tokyo, 3-8-1 Komaba, Meguro-ku, Tokyo 153-8902, Japan

**Keywords:** giant vesicle, oil-on-water droplet, water-in-oil emulsion, experimental evolution, microfluidic device, machine assisted experiment

## Abstract

Experimental evolution in chemical models of cells could reveal the fundamental mechanisms of cells today. Various chemical cell models, water-in-oil emulsions, oil-on-water droplets, and vesicles have been constructed in order to conduct research on experimental evolution. In this review, firstly, recent studies with these candidate models are introduced and discussed with regards to the two hierarchical directions of experimental evolution (chemical evolution and evolution of a molecular self-assembly). Secondly, we suggest giant vesicles (GVs), which have diameters larger than 1 µm, as promising chemical cell models for studying experimental evolution. Thirdly, since technical difficulties still exist in conventional GV experiments, recent developments of microfluidic devices to deal with GVs are reviewed with regards to the realization of open-ended evolution in GVs. Finally, as a future perspective, we link the concept of messy chemistry to the promising, unexplored direction of experimental evolution in GVs.

## 1. Introduction

As a complementary viewpoint to conventional biology that details the components based on the reductionism, constructive exploration for the common mechanisms that are fundamentally shared among living cells today has progressively developed. Hartwell and his colleagues pointed out that cell functions can be understood as a combination of modules and raised the importance tofo understanding the “design principle” of living cells by studying the mechanisms of interactions among such modules based on bioengineering and computer science [1]. For example, by re-designing the genetic circuit of *Escherichia coli*, Elowitz and his colleagues clarified the fact that the stochasticity of the gene expression is decomposed into intrinsic and extrinsic noise [2]. Constructing a simplified cell with minimal sets of genomes, i.e., minimal sets of functional modules, plausibly brings us an idea for the design principle of cells [3]. These approaches using today’s cells toward the design principle of cells are categorized as the “top-down” style, since the informational substances therein are involved into a central dogma. On the other hand, it is noteworthy to refer Langton’s pioneering statement “life as it could be”, which describes many types of possible life which may include ones far from what we know of in terms of life on the Earth [4]. Constructing cell-like system in “bottom-up” approaches, in which its components are assembled from scratch regardless of central dogma, will verify the uniqueness and certainty of the design principle of today’s cells [5].

In viewpoints of such bottom-up approaches, we postulate that, throughout life as it could be on the Earth (might be extended to this universe), life and its linkage have to be implemented evolutionarily. Therefore, the exploration of evolvable chemical systems with materials which are not necessarily related to today’s cells potentially includes a sufficient condition for emergence of life [5]. In other words, compared to the top-down approaches revealing the minimum requirements to maintain the life phenomenon of living cells, the constructive exploration for cell model in bottom-up approaches challenges to clarify the possible paths to reach to such a chemical system with the minimum requirements. 

The evolution of chemical systems can be deconstructed into two hierarchical stages. One is the evolution of constituent molecules (DNA, RNA, protein, and others), which is called as chemical evolution. With regards to chemical evolution, prior to the experimental approach, some theoretical studies have been performed for understanding how to maintain the autocatalytic reaction of RNA [6,7,8]. The other stage is the evolution of whole system including a molecular assembly. This type of evolution is more complicated than chemical evolution because it involves the evolution of interactions of modules. How do the modules, which are not necessarily related to each other, get together and constitute one system? Here we call this evolution of the whole system “modular association evolution”. Modular association evolution orients the complexity of the whole system and could become one of principal evolutionary structures in life phenomena. It is an extension of chemical evolution; it should be regarded as an example of open-ended evolution [9], following the assembly of modules composed of molecules. Note that the number of previous reports on this direction are extremely limited, and most of previous reports focused on the chemical evolution. It is certain that the chemical evolution continuously proceeds in the first place throughout the modular association evolution. We remark that chemical evolution could influence the process of modular association evolution, and thus is significant as a foothold to constructively aim at modular association evolution. 

Attempts to construct evolvable chemical systems are important not only for elucidating the fundamental mechanism of cells as mentioned above, but also for drawing out possible scenarios of the origin of life [10]. So far, various hypothetical scenarios, such as RNA World [11,12], Protein World [13,14], and Lipid World [15] have been proposed. In all stories, the establishment of a self-replication cycle is fundamental, and a chemical system functionalized while maintaining its stability as a whole system is hypothetically described. National Aeronautics and Space Administration (NASA) had raised the working definition of life as “self-sustaining chemical system capable of Darwinian evolution” for the exploration of extraterrestrial life [16]. However, nobody has revealed the components of such system and environments surrounding the system. Experimentally clarifying the possible scenarios of the origin of life may make it possible to concretize such a working definition of life and to make the precision and range of the exploration more realistic. From this point of view, it is desirable to construct an evolvable system in a wider context, not being limited to the body of cells living on Earth today. However, as a first step, a cell-like evolvable chemical system seems to be appropriate.

**Figure 1:**
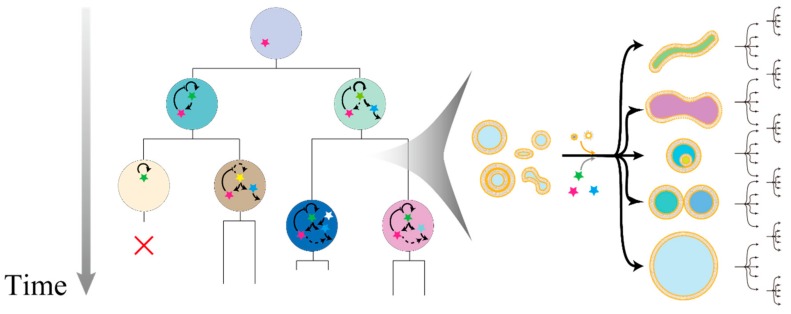
Hypothetical representation of an experimental evolution with giant vesicles (GVs) as a modular association evolutionary body. The inner components are modularly associated across generations, and these inner reactions are linked to their fitness or growth rate (**left**). The growth rate or “fitness” is dependent on deformation process in self-reproduction with diverse pathways partly because of the difference of the initial state, intake kinetics of membrane molecules and inner substances, and stochastic fluctuations (**right**).

The chemical cell models reported thus far are roughly classified into the following categories: water-in-oil emulsions [17,18] underwater oil droplets [19,20], oil droplets on water surface [21,22], and vesicles [23,24]. The advantage of emulsions and oil droplets lies in the simplicity of their components and the fact that these structures are easy to prepare. On the other hand, vesicles, which are closed bilayer membranes made of amphiphilic molecules in water (vesicles composed of phospholipids are called liposomes), have additional parameters to be considered, such as membrane fluidity, lamellarity, shape, compositional difference of inner and outer sides of the bilayer, and molecular sorting. However, the structure of vesicles most closely resembles that of the current cell. The important aspect is that both the inner and outer regions of vesicles are filled with water, and the inner phase is compartmentalized by a semipermeable membrane bilayer composed of amphiphilic molecules. Delivering substances to chemical cell models is indispensable to maintain the system steadily or possibly to expand the system to more complicated modules. Therefore, the advantage of the evolvable system based on vesicles is attributed to the ease of its interactions with the outer environment. Hence, it is plausible to choose vesicles as a starting module for cell models to scrutinize the dynamics of the assembly of other functional modules. In particular, a vesicle with a diameter of 1 µm or more, which is called a giant vesicle (GV), is desirable because of the similarity of its membrane structure and size to those of today’s cells. Therefore, the growth and division of GVs are vigorously studied to create self-reproducible cell models [25,26,27,28]. However, there are still no reports on GVs capabilities for stably repeating the self-reproducing cycle for long time periods ranging from tens to several hundred generations. Moreover, the coupling of inner functions and “fitness” remains unexplored (Figure 1). 

Since steady self-reproduction of GVs consists in the continuous transition of metastable states, long-time observation of individual GV is crucial to clarify the path dependency of GV deformation process under specific conditions. Moreover, variety of GV deformation requires us to analyze the observation result as a change of distribution. However, the compatibility of the precise observation and statistical analysis is still insufficient. Such technical difficulties must be overcome for understanding modular association evolution using GVs. Even when such an evolutionary system using GVs can be created, for exploring open-ended evolution with chemical cell models, experimental protocols with great efficiency are expected. Emerging techniques of laboratory automation for high-throughput methods have succeeded in cell experiments [29]. It is urgent to establish high-throughput experimental systems applicable to experimental evolution for GVs, and as its premise, methodology for accurately measuring GV dynamics should be developed. Recent developments of microfluidic device and their applications to GV experiments contribute to solve this issue. Hence, herein, we have reviewed and discussed the research trend of chemical cell models, emulsion, oil droplets, and vesicles, which have been applied for studying experimental evolution, together with the viewpoint of throughput, and summarized the recent progress of microfluidic technologies on GVs. 

## 2. Experimental Evolution with Water-in-Oil Emulsions

Experimental evolution in water-in-oil emulsions is mainly focused on chemical evolution processes, that is, evolutionary dynamics of encapsulated molecules [30]. These molecules are biomacromolecules such as RNA and DNA, which act typically as information molecules in today’s cells. With regards to biomolecular replicators, classical theoretical studies of chemical evolution have pointed out the problem of a replicator which is replicated by other catalysts and does not catalyze other replications. This replicator is called as a parasitic replicator. Error catastrophe [6], which decides the limitation of the length of information replicated with some errors, can be overcome by constructing an autocatalytic reaction network connected circularly (called as hypercycle) [7]. However, the hypercycle is vulnerable to parasitic species [8].

**Figure 2:**
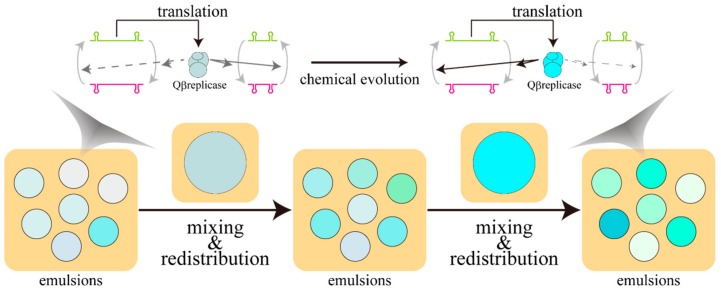
Schematic illustration of chemical evolution implemented in the water-in-oil emulsion. Well-reproduced sequences are prone to occupy the gene pool of the next generation. Strongly colored emulsions at the bottom part have a higher concentration of ribonucleic acid (RNA). Compartmentalization prevents the spreading of parasitic RNA species to the whole system, and the catalytic cycle can be maintained through the course of evolution. For visual readability, the color of Qβ replicase at the top was homologized to that of the emulsions at the bottom.

The first experimental findings on this problem were reported by Yomo’s group [31]. They encapsulated the reagents for the catalytic cycle of Qβ replicase, which is the RNA-dependent RNA polymerase of the Qβ phage, in the emulsions. After the performance of the catalytic cycle for a certain time period, the emulsions were collected, and part of the collected substance was again distributed into new emulsions. The proportion of the well-duplicated replicator of RNA in the next-generation emulsion becomes stochastically high, i.e., it can be understood as an experimentally implemented example of the genetic algorithm, a mathematical optimization algorithm resembling Darwinian evolution [32], using a combination of the emulsion and the catalytic cycle. RNA sequences responsible for the replication reaction were preyed upon by the parasitic RNA species in the bulk experiment. In contrast, in the case of emulsions, the increase of replication ability was observed against the parasitic RNA species (Figure 2). This can be attributed to the fact that the collapse of the system due to the parasitic RNA species is localized in one compartment of the emulsion so that their influence on the whole system can be suppressed. In other words, the significant role of compartmentalization in the chemical evolution of the replicator was proven experimentally for the first time. It should be noted here that the introduction of selection pressure for RNA sequences was carried out for the entire system by collecting and redistributing the emulsions. 

On the other hand, Griffths’ group reported another experimental chemical evolution with Qβ replicase in emulsion using a microfluidic device [33]. They inserted trans Varkud satellite ribozyme into the minus strand, which catalyzes the cleavages of fluorescent quencher-linked RNA sequences from reporter RNA. Therefore, only emulsions containing the full-length RNA species can be fluorescently detected. Water-in-oil droplets generated inside the microfluidic device were selected by a fluorescent-activated droplet sorting technique [34]. The collected emulsions were united and redistributed into the next generation. The microfluidic device has an important role to enable the homogeneity and accurate control of experimental parameters, such as concentration, droplet size, sorting parameters, and so on, for each emulsion. It is also noteworthy that using the microfluidic device has made it possible to operate each compartment with direct observations in the statistical scale, comparable to the fluorescence-activated flow cytometer. As a result, only under the selected and compartmentalized condition, RNA sequences having the fluorescent catalytic ability did not become extinct in the ninth generation. Under the unselected compartmentalized condition, time scale to the extinction was slightly slowed down, but was not enough to avoid the extinction of catalytically active RNA. The most significant difference of this study from the former study reported by Yomo’s group was that the functional feature (=fluorescent catalytic activity) was not necessarily related to their own replication ability, but was constructively coupled to the viability of the individual (=emulsion).

## 3. Experimental Evolution with Oil-on-Water Droplets

When one adds oil to water, oil droplets are formed, and in the presence of surfactants, some oil droplets migrate autonomously. These self-propelled oil droplets are roughly classified into oil-in-water droplets [19,35,36] and oil-on-water droplets [21,37]. The mechanism of these migration phenomena is thought to be based on the Marangoni effect [38] which occurs in the relaxation process on the asymmetric interfacial tension due to the unevenly adsorbed surfactant on the oil droplet interface [19,21]. The detailed manner of this migration strongly depends on the compositions of the oil and surfactant solution; this can be interpreted as an example showing that the composition itself can act as the information of traits without having an explicit information molecule such as DNA or RNA. This overlaps with the evolutionary blueprint in a theoretical model in which the components of the self-assembly itself are used as information (GARD model) [39]. Therefore, optimizing the composition of molecular assembly is also in the scope of experimental evolutions.

**Figure 3:**
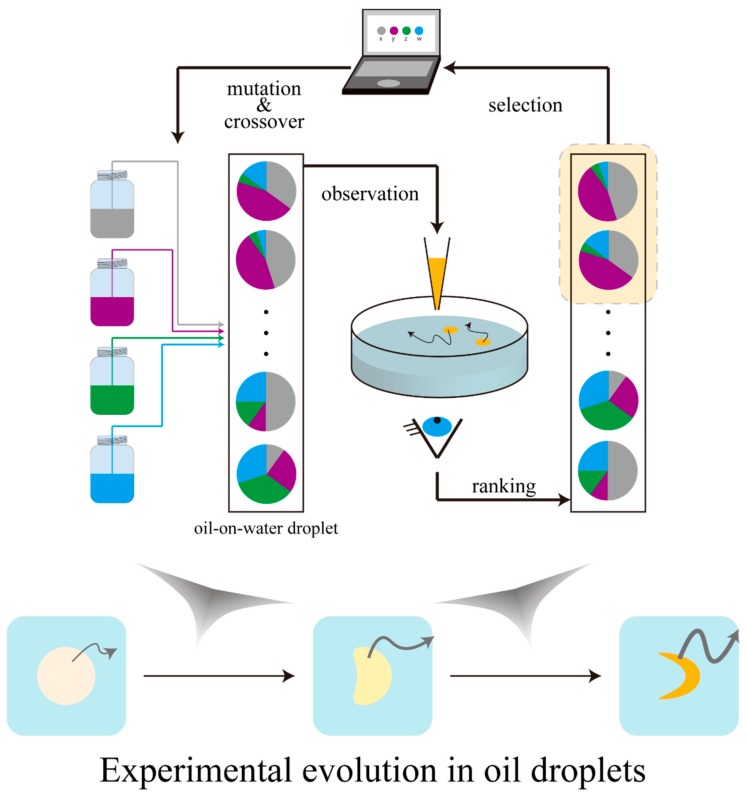
Schematic illustration of the evolutionary cycle of water-on-oil droplets. Oil droplets are ranked based on the observation, and the “gene” pools of the next generation are computationally constructed based on the high-ranked compositions. Oil droplet movements become sophisticated with the passing of generations, according to their compositions.

Cronin’s group has performed systematic research on the evolution of oil-on-water droplets with diameters of the millimeter to centimeter order (Figure 3) [40,41,42]. The distinguishing feature of oil-on-water droplets as a strategy to study experimental evolution is the ease to control the composition (genotype) and to define phenotype based on the observation. Mixture ratios of four types of oil (1-octanol, diethylphthalate, 1-pentanol, and octanoic/dodecanoic acid) were assumed as the genotypes, and migration modes classified into 9 types were interpreted as the phenotypes. They defined “fitness” according to a specific mode of motion (division, mobility, and vibration) and ranked the compositions of the droplets to construct a next-generation “gene pool” with mutations and crossover. These experimental procedures were implemented in an automated manner. Using machine-assisted systems made it easy to search for promising experimental parameters such as the mixing ratio of the four oils prior to the experimental evolution. As a result, the mean fitness observed for each generation gradually rose, which suggested the evolution of the oil droplets. It is noteworthy that by implementing the selection as a machine-assisted experimental system, long-term experimental evolution for more than twenty generations was achieved. Recently, by using a 3D printer, the group has constructed an experimental system that scopes the adaptation phenomenon of self-propelled oil droplets to pseudo environmental changes by the arbitrary modelling of the space in which the self-propelled oil droplets can migrate [41].

The evolution of oil-on-water droplet by Cronin’s group is, to the best of our knowledge, the only experimental example which focused on the evolution of the compartment linked to the observable phenotypes. However, the low compatibility for water-soluble or biological macromolecules in the oil droplets causes a limitation for further exploration on chemical cell models toward modular association evolution. 

## 4. Experimental Evolution with Vesicles

Examples of tracking evolutionary dynamics of vesicles are even rarer. For example, experimental evolution in a pore-forming membrane protein, α-hemolysin, was reported using a fluorescence-activated flow cytometer to impose selection pressure [43]. The production (or the permeation efficiency of a membrane protein itself) of α-hemolysin can be evaluated spectroscopically by measuring the incorporation efficiency of a fluorescent substrate from the external aqueous phase. By sorting highly fluoresced liposomes with the fluorescence-activated flow cytometer, the evolutionary cycle for the DNA sequence inside the liposome was constructed. The method of displaying the products generated in the liposome to impose the selection pressure is called the liposome display method [44]. According to this method, the time required for one round is around 24 h, and it is reported that the final desired sequence can be obtained in 1–5 weeks. Nemoto’s group reported another example of peptide evolution using liposomes [45]. By using Puromycin-linker, the translated peptide and their original cDNA were linked together after reverse transcription. They constructed first-generation DNA pools with intentional bias to produce arginine-rich peptides to promote their interaction with the liposomal membranes. cDNAs, which are translated into membrane-philic peptides, were collected by centrifuging the mixed liposome dispersion, and they would be used for the second-generation DNA pools. Interestingly, peptides that are toxic to the membrane were autonomously expelled during this centrifugation process. Therefore, peptides were potentially compelled to bind to the membranes more strongly. 

These two studies focused on chemical evolution, but it is possible to interpret the evolution of such membrane-philic peptides as an evolution of compartment module. For example, Noireaux and Libchaber succeeded in the construction of α-hemolysin expressing liposome and demonstrated that the expression of enhanced green fluorescent protein (eGFP) therein was sustained by the α-hemolysin because of the high permeability of substances from outer solution [46]. It implies that there is an optimized state of compartment module (membrane embedding α-hemolysin) for the robustness or high functionality of inner reaction networks (gene expression of eGFP), and vice versa. Thus the evolution of compartment module can be linked to an experimental evolution of inner modules in future.

## 5. Development of Handling Techniques of GVs Using a Microfluidic Device

Let us review the advantage of using GVs for constructing a modular association evolution system. Considering the chemoton, the most basic unit of evolution advocated by Gánti [47], the production of compartment molecules is explicitly assumed. The production of components requires the supply of the substrates from the outer environment. The commodity of the inner phase and the outer phase is helpful to cater the molecules for the chemical model from the external environment with higher degrees of freedom (in principle) compared to emulsions and oil droplets. In this sense, vesicles are the best agents for experimentally validating this theoretical model. Of course, the ease of delivering substances to vesicles is advantageous for studying modular association evolution. Furthermore, in case of GVs, the direct observation of deformation, which is inevitable for self-reproduction, is possible, and when one visualizes the internal state with a fluorescent probe, it is possible to examine the relationship between the dynamics of the compartments and inner reactions with high resolution. However, to fully exploit these potential advantages such as individual measurement capability and the responsiveness to the external environment, development of a highly accurate and flexible observation technique for one GV would be prerequisite.

**Figure 4:**
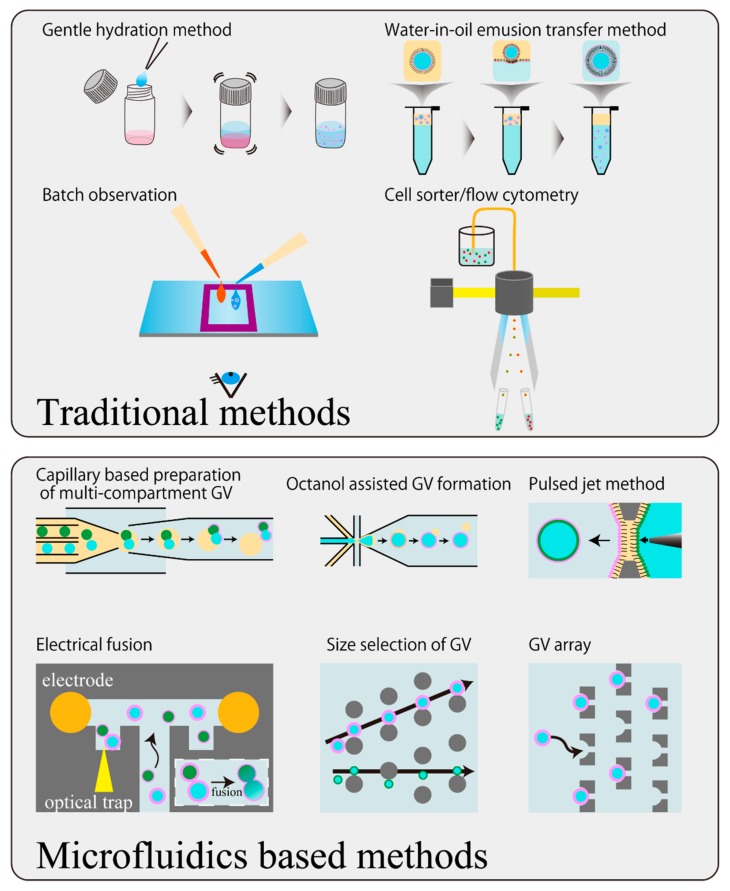
Schematic illustration of updating the manipulation techniques of GVs by microfluidic technology. The gentle hydration method and water-in-oil emulsion transfer method are illustrated as examples of traditional GV preparation methods, and batch observation and flow cytometer are depicted as examples of traditional observation methods.

Currently, microfluidic devices are promising for meeting these requirements (Figure 4). For example, with regards to the preparation method of GVs, a device which performs the conventional electroformation method on a chip has been reported [48]. With regards to the water-in-oil emulsion transfer method, a device that makes the emulsification process highly accurate with high throughput has been reported [49]. Recently, a capillary-based device that replaces all the processes of the transfer method in one chip has been reported [50,51]. The device further enables us to prepare vesosomes (vesicle in vesicle), which are difficult to produce precisely in conventional studies. One problem of the emulsion-based preparation method would be the residual oil in the prepared GVs. A device implementing the mechanical removal of the oil phase following phase separation has been reported [52] based on the octanol-assisted GV formation method [53]. Combining these devices and a pico-injection device which electrically injects inner substances in the droplet with controlled volume for emulsion [54], would solve a considerable part of the technical problem concerning the degree of freedom of the initial internal water phase of the GVs. A sophisticated application of microfluidic technology for precise GV formation with highly controlled inner water phase was recently developed by Spatz’s group [55]. They constituted GV from small vesicles in emulsion droplets surrounded by polymer monolayer to bestow mechanical stability, and the GVs were applied to the pico-injection device. This group confirmed that the polymer shell can be removed after pico-injection and succeeded in the preparation of GVs encapsulating biomacromolecules such as actin and microtubles. Meanwhile, a method capable of accurately producing GVs with the asymmetric outer and the inner leaflets of the membrane, called the pulsed jet method, has been proposed [56].

As a device for controlling the dynamics of GVs, there is an example of an experimental platform that can generate the fusion and division of vesicles by electrical stimulation using electrodes [57]. In addition to this, several fusion-inducing devices for GVs have been reported [58,59]. Alternatively, a device in which mechanical division with obstacles was constructed in a hypertonic solution was reported [60].

Devices specialized for observing the individual dynamics of GVs have also been developed, for example: a multilayered device utilizing branching of flow in which the height of the ceiling is partly lowered [61], a device that captures GVs following to the production in a single chip [48], a device capable of blocking the flow field by using a surrounding ceiling switchable by pneumatic pressure [62], and a device in which GVs of only a limited size are trapped by combining size selection and arraying structures [63]. By capturing and arraying in this manner, it is possible to completely exchange the external solution while directly observing each GV. This in turn makes it possible to evaluate the behavior of GVs, like deformation, with the evaluation of the variation for each GV exposed to chemical stimuli such as osmotic pressure and supplying membrane molecules.

## 6. Summary and Future Perspectives

From the viewpoint of constructing evolvable chemical cell models, we have focused on the techniques for studying experimental evolution including machine-assisted experiments, and the recent studies on water-in-oil emulsions, oil-on-water droplets, and vesicles have been summarized. In these works, various machines, including a fluorescence-activated flow cytometer, microfluidic selection device, home-made machine-assisted platform, and so on, were indispensable for examining experimental evolution. In other words, the chemical system was extended by the machines for implementing selection pressure. As a result, fitness, or direction of evolution, had been explicitly defined in advance when the experiments were designed. The open-ended experimental evolution would be the next unexplored field. The most significant point to experimentally explore this aspect is how the functional fitness is implemented after being linked to the self-replication process. This is directly related to the scope of the modular association evolution: how does the cell become modularly complicated? In other words, what is the design principle of the cell? The report from Griffith’s group implied a preliminary example of this nontrivial relationship between the functions and the replication ability of the cell models.

Self-reproducing GVs are expected as promising agents for studying modular association evolution. Since the self-replication of GVs occurs on a mesoscopic scale, it is partly separable from the other inner functions. In other words, in the self-reproducing GVs, the replication mechanics can be extracted as one of the modules of the whole system. It guarantees a room for the redundancy of the other functional modules. Importantly, however, all the modules are spontaneously linked to the fitness of GVs, with relation to division dynamics of compartment. Several examples of the construction of self-reproducing GVs have already been reported [25,26,27,28]. Exploring the delivery of substances to the inner aqueous phase has also begun. For example, pioneering work to construct the gene expression system of α-hemolysin in GVs was reported by Noireaux et al. [46]; additionally, in recent years, Adamala and her colleagues have reported modularized logic gates by utilizing vesicles exchanging inner substances [64]. Although GVs have the potential advantage as discussed above, the following technical issues remained difficult to be solved: the manufacturing method and the external condition had to be ad hoc, as there was no high-precision direct observation method in a statistical scale. However, as we have reviewed in this manuscript, the development of microfluidic device in recent years has considerably mitigated these issues associated with GV-based experiments.

What is a next step of the experimental evolution for modular association evolution? We here bring up a question related to open-ended experimental evolution. In the process of open-ended experimental evolution, the environment surrounding GVs should not be designed specifically for the experimenter’s intension. This unintended attitude on experiments might be linked to the basic concept of messy chemistry [65,66]. Messy chemistry investigates chemical systems with high diversity of products, intermediates, and reaction pathways as the whole complex system expecting emergent dynamics, such as origin of life. It is a concept opposed to traditional “clean” chemistry, which focuses on one or at most, several well-identified reactions. The border between messy and clean chemistries is determined by the current analytical technologies and acknowledges about the objects. When we describe the close-up of experimental evolution of GVs regarding to the concept of messy chemistry, the environment surrounding the GVs should not be optimized for the myopic purposes in the experimental protocols. However, well-controlled experimental environment will be inevitable when the knowledge of the dynamical relationship between the stimulation and response of GVs is in the nascent stage. Importantly, the recent development of microfluidic devices will serve as the corner stone for such studies. To fully relish the benefits of the studies on this direction, exhaustive studies are becoming increasingly important. The search for promising parameter spaces beforehand by the automation of experimental settings has already been accomplished partially for studies regarding experimental evolution using oil-on-water droplets. In addition to that, in the future, a mathematical approach, such as the Bayesian optimization method, would be promising to explore the vast parameter space more efficiently. With regards to cell biology, in which basic manipulation protocols such as cultivation and stimulus application are well established, successful examples of experimental evolution studies have been reported recently [67,68]. Moreover, the integrated environmental controlling system has already begun to be established and been made commercially available by pioneer venture companies, such as Berkeley lights, Inc. (Emeryville, CA, USA) [69]. Therefore, to explore the experimental evolution with plausible chemical cell models, integrated and automated microfluidic device systems for the preparation, observation, and stimulus application of GVs are becoming increasingly important.
